# Quantifying single-cell secretion in real time using resonant hyperspectral imaging

**DOI:** 10.1073/pnas.1814977115

**Published:** 2018-12-10

**Authors:** José Juan-Colás, Ian S. Hitchcock, Mark Coles, Steven Johnson, Thomas F. Krauss

**Affiliations:** ^a^Department of Physics, University of York, Heslington, YO10 5DD York, United Kingdom;; ^b^Department of Electronic Engineering, University of York, Heslington, YO10 5DD York, United Kingdom;; ^c^Department of Biology, University of York, Heslington, YO10 5DD York, United Kingdom;; ^d^Kennedy Institute of Rheumatology, University of Oxford, Headington, OX3 7FY Oxford, United Kingdom

**Keywords:** single-cell analysis, label-free, photonic biosensing, photonic crystal

## Abstract

Cell communication is primarily regulated by secreted proteins, whose inhomogeneous secretion often indicates physiological disorder. Parallel monitoring of innate protein-secretion kinetics from individual cells is thus crucial to unravel systemic malfunctions. Here, we report a label-free, high-throughput method for parallel, in vitro, and real-time analysis of specific single-cell signaling using hyperspectral photonic crystal resonant technology. Heterogeneity in physiological thrombopoietin expression from individual HepG2 liver cells in response to platelet desialylation was quantified demonstrating how mapping real-time protein secretion can provide a simple, yet powerful approach for studying complex physiological systems regulating protein production at single-cell resolution.

## Significance

Cell communication is primarily regulated by secreted proteins. However, inhomogeneous secretion may indicate commencement of disease. Therefore, the ability to parallelly monitor innate individual cell-secretion kinetics is crucial to understand systemic malfunctions. Here, we report a high-throughput method for parallel, in vitro, and real-time analysis of specific single-cell signaling using hyperspectral photonic crystal resonant technology without the need of adding any fluorescent label. We mapped the secretion of a signaling protein (thrombopoietin, TPO) from individual human HepG2 cells and quantify the heterogeneity in TPO expression as a function of the desialylated (aged) platelet level concentration. Given its ease of use and implementation, we anticipate that our technology will transform single-cell protein expression analysis for applications beyond basic research.

Proteins that are secreted from cells into the extracellular matrix make up 13–20% of the entire human proteome (1). These secreted proteins typically function as mediators of cell–cell signaling and cellular proliferation and thus play an important role in a wide variety of important physiological and pathological processes. A prime example of this is hematopoiesis, which is tightly regulated by the paracrine or endocrine secretion of cytokines and hormones to maintain hematopoietic stem cells and drive specific lineage differentiation. Impairment of this regulatory system, through organ failure or deleterious mutations, can result in malignant overproduction of blood cells, aberrant immune function, or bone marrow failure.

Current understanding of secreted proteins is based largely on traditional proteomic analysis, such as ELISAs and mass spectrometry (MS), of proteins in the extracellular medium. While these techniques provide the high sensitivity required to detect low concentrations of proteins secreted into the surrounding matrix, a comprehensive understanding of protein secretion also requires tools with sufficient resolution to map the temporal and spatial regulation of secretion. For example, cytokine secretion by innate immune cells is orchestrated between cells to provide an initial inflammatory or allergic response and later to ensure this response subsides in a timely and coordinated fashion. Traditional ELISA and MS both lack the spatial and temporal resolution required to map the kinetics of such tightly regulated and orchestrated protein secretion. Furthermore, single-cell analytical techniques have shown that individual cells with apparently identical phenotype exhibit heterogeneity in protein secretion. Thus, the ability to interrogate and monitor biological systems at the single-cell level provides a precise route for determining which variation is random and which is meaningful, and for building better models of the behavior of individual cells. Again, ELISA and MS typically measure the average response of a cell population at a fixed time point and are thus unable to screen the “hidden world” beneath population averages (2). Robust and nondisruptive methodologies to profile protein secretion at the single-cell level and to map changes in secretion due to cooperative interactions or exposure to therapeutics are critical if we are to fully understand their role in both healthy and diseased states.

Spatial confinement provided by microfluidic systems has been exploited for molecular analysis and the single-cell level and subnanoliter microwell arrays have recently been employed to analyze protein secretion at single-cell resolution (3). Here, protein detection was achieved using a sandwich immunoassay with a fluorescently labeled detection antibody in which the fluorophore was excited by total internal reflection microscopy. While providing insight into the kinetics of protein secretion from single cells, the binding of the detection antibody to form sandwich immunocomplexes is the rate-limiting step, ultimately limiting the temporal resolution of this approach. Furthermore, the need to spatially confine single cells within individual microwells precludes the possibility of mapping protein secretion in cellular communities in which protein secretion is orchestrated between interacting cells. More recently, an optofluidic approach based on nanoplasmonic sensing has been proven as a specific and sensitive technique to measure cytokine secretion at the single-cell level (4). While overcoming the need for a detection antibody due to its intrinsic ultrasensitivity (detection limit in the pg/mL range), this spectroscopic approach is designed to report protein secretion from an isolated single cell; thus, is not able to map orchestrated protein secretion in cellular communities or provide high-throughput analysis of secretion dynamics. Similarly, McDonald et al. (5) demonstrated an alternative approach for the real-time detection of secreted proteins based on interferometric detection of scattered light (iSCAT). While this removed the limitations imposed by fluorescently labeled antibodies and microfluidic confinement, the lack of immunoreagents limits the specificity of the approach; iSCAT is sensitive to individual proteins with molecular weights in the range 100–1,100 kDa, but lacks specificity to differentiate between proteins within this range. Furthermore, the field of view was here limited to only 6 μm, again unable to report parallel secretion from single cells within large populations.

Here, we present a simple, yet powerful technology to monitor and quantify orchestrated cell signaling at the single-cell level, in real time and in a label-free manner using photonic crystal resonant imaging. Photonic crystal structures, including 1D gratings, have recently gained considerable attention as they offer the same evanescence wave sensing principle (6) and performance of surface plasmon resonant sensors without the need of complex instrumentation, e.g., bulk prism-coupling instrumentation, allowing further integration with diverse laboratory-based apparatus, such as phase contrast and fluorescent microscopy. Photonic crystal resonant surfaces (PCRS) consisting of silicon nitride (Si_3_N_4_) or titanium dioxide (TiO_2_) have been demonstrated that allow label-free cellular imaging with subcellular spatial resolution (resolution 2–6 µm depending on the device orientation) through resonant wavelength-based hyperspectral imaging (7) (*Methods*). Essentially, PCRS exhibit a spatially confined resonant wave at specific wavelengths, which interferes constructively or destructively with the incoming light beam, leading to a minimum (maximum) in the transmission (reflection) spectrum (*SI Appendix*). Moreover, PCRS can be fabricated over large areas to provide a high field of view (the order of centimeters), to allow parallel imaging of multiple cells without affecting the intrinsic spatial resolution.

**Fig. 1.**
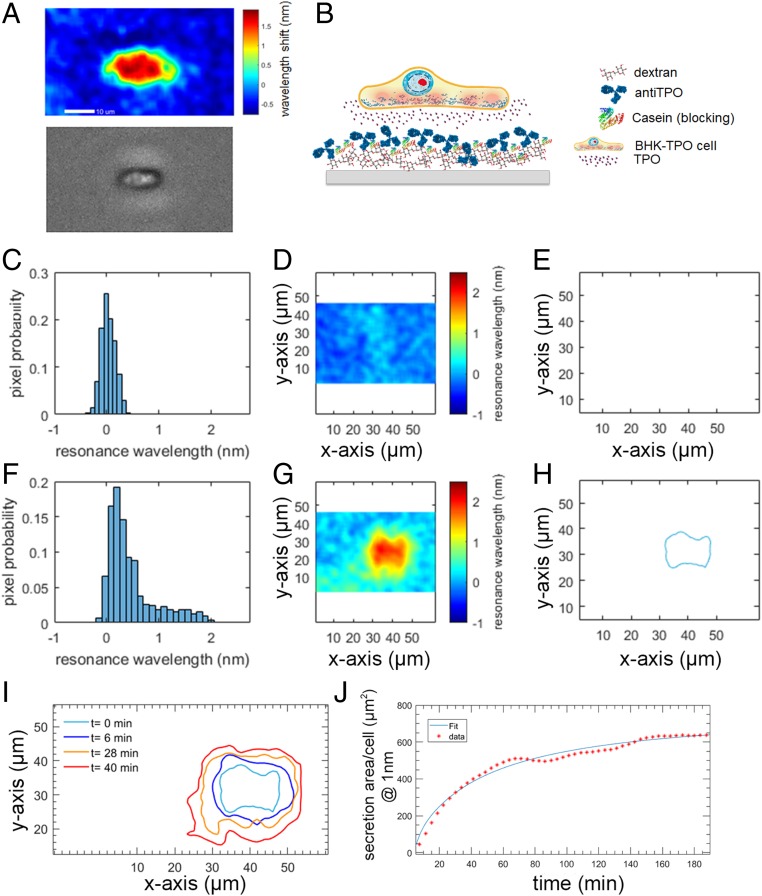
Photonic crystal resonant imaging protocol for monitoring TPO secretion from a single BHK-TPO cell. (*A*) Comparison between a photonic crystal resonant-imaging hyperspectral image (*Top*) and a phase-contrast microscope image (*Bottom*) of a single BHK cell attached to a PCRS. Higher-resonance wavelength shifts observed using PCRS are related to higher refractive index values, originating from the presence of both the cell and the secreted biomolecules. Secreted molecules are transparent and cannot be resolved by phase-contrast microscopy. (*B*) Dextran molecules are employed to create a 3D network of antibodies. Casein protein is employed as a blocking agent to prevent nonspecific binding from other proteins expressed by the cells. The secreted cytokines (i.e., TPO) from attached cells diffuse through the 3D network to specifically bind to the antibodies immobilized throughout the dextran network. The amount of deposited casein protein is optimized to maximize the signal-to-noise ratio of the detection system. (*C*) Gaussian distribution of the fraction of resonant pixels over the defined region of interest before BHK-TPO immobilization. Here the width of the Gaussian distribution was ±0.5 nm and the mean was set to 0 nm. (*D*) The region of interest (55 × 40 µm), with a wavelength uniformity of Δλ ± 0.5 nm. (*E*) No content is observed for the chosen threshold value of Δλ > 1 nm, indicating that no cell is present. (*F*) Upon attachment of the cell to the surface, the distribution of the pixel probability of the resonance wavelength over the defined region of interest increases to −0.2 < Δλ < 2.2 nm, indicating the presence of biomolecular content associated with the presence of both BHK-TPO and secreted TPO. (*G*) A hyperspectral PCRS image reveals the adhesion of a -BHK-TPO cell to the PCRS, whose high concentration of cell adhesion molecules located in the inner region is translated into a higher refractive index content area. (*H*) By setting a threshold of 1 nm from the central resonance wavelength, a contour plot is obtained representing the area defined by the adhered BHK-TPO cell. (*I*) Over time, this secretion area increases as TPO molecules are secreted from the cell and bind to the surface-immobilized antibodies, therefore locally increasing the refractive index around the BHK-TPO area. (*J*) The secretion of TPO is then monitored over time, and a Langmuir adsorption distribution is fitted to the data to model the secretion from the BHK-TPO cell accounting for the area covered by the adhered cell.

As the evanescent field is confined close to the grating surface (penetration depth ∼ 200 nm), the sensors are highly sensitive not just to large refractive index changes as a consequence of cell attachment to the sensor surface (Fig. 1*A*), but also to smaller, local refractive index changes such as those originating from protein–ligand binding events occurring on the sensor surface, all in real-time, label-free, and a quantifiable manner (8). By functionalizing the photonic crystal sensor surface with capture molecules (e.g., antibodies), the surface can thus be rendered sensitive to specific biomolecules secreted by the cells, enabling mapping of protein-secretion gradients at the single-cell level without the need of a fluorescent label. Instead of immobilizing antibodies such that they are ordered on a single plane on the sensor surface, we increased the density of antibodies within the evanescence wave of the PCRS, and thus the detection sensitivity, by creating a 3D network using branched glucan dextran (*Methods*). The surface was finally treated with purified casein to inhibit nonspecific binding of other molecules secreted into the extracellular matrix (*Methods* and *SI Appendix*) (Fig. 1*B*). This blocking approach allowed us to maximize the signal-to-noise ratio of our detection system and perform long-term analysis of cellular activities under appropriate conditions.

To demonstrate the performance of our technology, we first mapped the secretion of thrombopoietin (TPO) from an immortal cell line comprised of baby hamster kidney cells (BHK). TPO is a hematopoietic cytokine essential for driving the differentiation of megakaryocytes and maintaining physiological levels of circulating platelets. Additionally, TPO is one of only a small number of cytokines responsible for maintaining hematopoietic stem cells. The TPO-secreting BHK cells [BHK-TPO (9)] used here constitutively release TPO without the need for an extracellular triggering biomolecule (*Methods* and *SI Appendix*). The BHK-TPO cells are also adherent, and assemble in a monolayer on the sensor surface, which here was functionalized with antibodies selective against TPO (*Methods*) to map the secretion of TPO from individual BHK-TPO cells. A resonant image of the PCRS before cell adhesion (i.e., PCRS functionalized with dextran, anti-TPO antibodies, and casein) was recorded (Fig. 1*D*) over a 55 × 40-µm region of interest and the fraction of pixels resonant at a specific wavelength plotted on a histogram (Fig. 1*C*). The mean resonance wavelength of the functionalized PCRS was subsequently fixed to 0 nm to provide a baseline resonant image. The width of this Gaussian distribution (±0.5 nm) is largely a consequence of inhomogeneity in the surface chemistry plus experimental error and thus a threshold of 1 nm was set. Shifts in the resonance wavelength above this threshold arise due to local changes in refractive index due to binding of BHK-TPO cells/TPO to the surface.

The functionalized PCRS were subsequently exposed to BHK-TPO cells suspended in the supporting culture media [concentration of 10^5^ units per milliliter (U/mL), significantly below the 100% confluence level to avoid contact inhibition of inherent protein/gene expression] and the adhesion of a single BHK-TPO cell in our defined region of interest was recorded using hyperspectral imaging. The local increase in refractive index associated with the adhesion of a single cell to the PCRS induced a change in the resonance wavelength over the region of interest (Fig. 1*G*) which again can be quantified by the histogram of the fraction of pixels resonant at a specific wavelength (Fig. 1*F*).

**Fig. 2.**
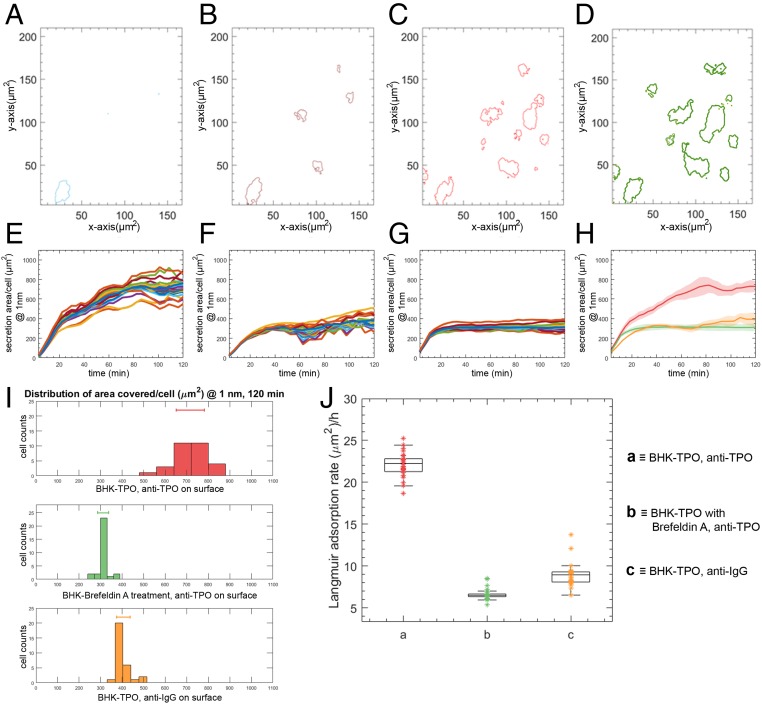
Single-cell dynamics analysis of a 30 BHK-TPO cell population. (*A*–*D*) Hyperspectral contour plots (at a threshold of 1 nm above the average resonance wavelength) at 0, 8, 15, and 23 min, respectively, of a 160 × 210-µm region of interest where BHK-TPO cells attach to the functionalized PCRS. (*E*) Individual traces of the real-time secretion area coverage of a 30-cell BHK-TPO population on a TPO specific functionalized surface. The robustness and the versatility of our method was further demonstrated by challenging the PCRS with two different 30-cell population control systems: (*F*) firstly with a system in where the surface was rendered IgG selective (the fluctuation over time is attributed to the accumulation of nonspecific interactions between the secreted TPO and immobilized IgG antibodies), and (*G*) secondly with a BHK-TPO population treated with the protein transport inhibitor brefeldin A. (*H*) The real-time response from the three systems over 130 min yielded notable and quantitative differences. The direct system (BHK-TPO with a TPO-selective PCRS functionalization, in red) exhibited an average secretion area coverage Γ_anti-TPO_ ∼ 720 µm^2^, while this average secretion area coverage was nearly reduced to a half in the two control systems [Γ_anti-IgG_ ∼ 400 µm^2^ (orange), Γ_brefeldin__A_ ∼ 300 µm^2^ (green)], indicating the specificity and robustness of both assay and extraction method. The shaded areas represent the SD for each trace at that time instant. (*I*) We analyzed the heterogeneity within each 30-cell population by studying the SD in secretion area coverage distribution at 120 min, which revealed an ∼2-3-fold higher secretion area in the direct system over the two control systems. (*J*) We modeled the Langmuir rate constant associated with each system to deconvolute the influence of the inherent cell attachment area from the measurement. We obtained rates of ∼22, 9, and 6.5 µm^2^/h for the direct, nonspecific, and nonexpressing systems, respectively.

Subsequently, resonant images of the region of interest collected at 3-min time intervals showed a shift in the resonance frequency around the periphery of the immobilized cell, corresponding to an increase in the local refractive index, the spatial extent of which increased with time. This can be seen in the contour plot of Fig. 1*I* (also see *SI Appendix*), which shows the spatial position over the region of interest at which the resonance wavelength increases beyond the 1-nm threshold. This increase in resonant frequency around the cell is associated with the local increase in refractive index due to TPO secreted from the BHK cell binding to the anti-TPO antibodies immobilized on the PCRS. This is further confirmed by the absence of a shift in resonant frequency in control experiments where the surface was functionalized with an antibody which lacks specificity to TPO (here, the surface was functionalized with antibodies specific for IgG) or when using BHK-TPO cells in which the TPO secretion has been suppressed using the protein transport inhibitor brefeldin A (Fig. 2 *F* and *G*).

To aid comparison between experiments and quantify the kinetics of protein secretion from a single cell, we define the total secretion area covered by the cell and surface bound TPO (i.e., the area equivalent to a shift in resonant frequency 1 nm above the background resonance). To further calibrate the measurement, we subsequently subtract the region relative to cell attachment from this total secretion area, which is statistically delimited by a shift in resonant frequency of 1.7 ± 0.1 nm (*SI Appendix*). Fig. 1*J* shows the change in total secretion area covered by the contour plot as a function of time. Assuming the secreted TPO binds to the surface-immobilized antibody as a single adsorption process, the time evolution of this secretion process can be modeled as a Langmuir adsorption distribution yielding a single-cell secretion rate of 22 µm^2^/h (coefficient of determination R^2^ of 0.94 ± 0.04) (*Methods*).

The ability to image and map protein secretion from multiple single cells is critical to understanding heterogeneity of cell populations. The wide field of view provided by our technology enables multiple, parallel measurements of single cells in real time to provide insight into such complex cellular processes. We extended the region of interest to an area of 500 × 500 µm (0.25 mm^2^) to characterize the dynamics and the kinetics of a population of 30 individual BHK-TPO cells without compromising our subcellular spatial resolution (7). We employed the same protocol described previously for a single BHK-TPO cell to quantify protein secretion from multiple cells within the new region of interest. A threshold of 1 nm was again chosen to define the background resonance and the increase in resonance around each cell was monitored every 3 min for a duration of 130 min (Fig. 2*E*; note for clarity a region of interest of 160 × 210 µm is shown at time points 0, 8, 15, and 23 min). To demonstrate the specificity of our assay we also challenged our PCRS sensor with two different control systems. In the first control, the PCRS was functionalized with a control antibody and challenged with BHK-TPO cells (Fig. 2*F*). The second control comprised a surface functionalized with anti-TPO challenged with a population of BHK-TPO cells treated with the protein transport inhibitor brefeldin A to suppress secretion of TPO (Fig. 2*G*) (*Methods*). Secretion of TPO and subsequent binding to surface immobilized anti-TPO led to an increase in the resonant frequency around each of 30 BHK-TPO cells (Fig. 2*A*). After 120 min, TPO secreted from the BHK-TPO cells had diffused isotropically away from each cell over an average secretion area of Γ_anti-TPO_ ∼ 720 µm^2^ around each cell. In contrast, the average shift in resonant frequency around individual cells in the control experiments was minimal due to the inhibition of TPO secretion from BHK-TPO cells treated with brefeldin A and the lack of specificity of the anti-IgG sensor for TPO (Fig. 2*H*).

The wide field of view coupled with high spatial resolution allows statistical analysis of protein secretion from individual cells. Histograms of surface adsorption for each BHK-TPO cell (Fig. 2*I*) revealed a high degree of heterogeneity in terms of TPO secretion (SD of Γ_anti-TPO_ ∼ 120 µm^2^). This contrasts the control system treated with brefeldin A which exhibits a lower SD (Γ_brefeldin__A_ ∼ 60 µm^2^) due to the homogeneity of the nonsecreting system. The heterogeneity of protein secretion was also observed in the kinetics of TPO secretion (*Methods* and Fig. 2*I*). The average rate of protein secretion from BHK-TPO cells and adsorption onto surface immobilized anti-TPO was κ_anti-TPO_ ∼ 22 µm^2^/h (coefficient of determination R^2^ of 0.94 ± 0.04), significantly higher than the kinetics of nonspecific adsorption observed in the control systems (∼9 µm^2^/h and ∼6.5 µm^2^/h for κ_anti-IgG_ and κ_brefeldin__A_, respectively).

The real-time and label-free capabilities associated with our analytical technology enable not only high-throughput analysis at the single-cell level but also the ability to study single-cell-dynamics in clinical samples, for example the personalized response of patients’ cells to drugs or other treatments. To highlight this capability, we have used this technology to determine the effects of human platelets on mediating TPO release at the single-cell level. Maintaining physiological levels of circulating platelets relies on a finely tuned balance between platelet generation and clearance. Although TPO is the key regulator of platelet production, the specific mechanisms that underpin TPO production and this physiological feedback loop remain unclear (10–12). Recent experiments suggest that TPO levels are directly dependent on the concentration of circulating, desialylated (aged) platelets (13). However, the levels of TPO protein up-regulation and the kinetics of TPO secretion at the single-cell level have yet to be quantified. We have thus applied our method to answer these important questions.

**Fig. 3.**
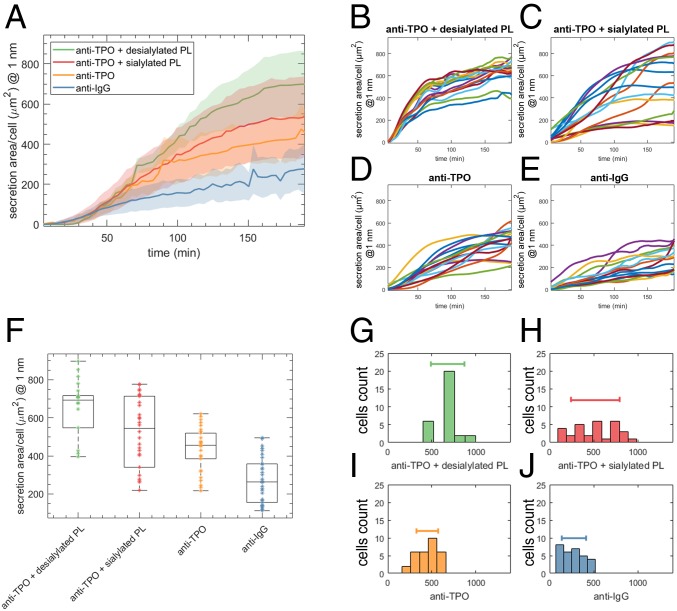
Levels of desialylated platelets affect TPO expression from HepG2 cells. (*A*) Real-time TPO secretion from a population of 30 HepG2 cells using hyperspectral PCRS imaging. Average TPO secretion from HepG2 cells cultured in 1,000 platelets per cell media with 99% desialylated platelets is 1.5 times× than the inherent TPO (*Methods* and *SI Appendix*). The shaded areas represent the SD for each trace at that time instant. (*B*) Traces within the 30-HepG2 cell population exhibit a small SD in the area of secretion of Γ_anti-TPO,−sia_ ∼ 320 µm^2^. (*C*) In contrast, the HepG2 cell population cultured in 1,000 platelets per cell media with 8–9% desialylated platelets shows an SD of Γ_TPO,+sia_ ∼ 600 µm^2^, indicating a high degree of heterogeneity due to unsynchronized and uneven TPO expression within the population with respect to the system shown in *B*). (*D* and *E*) Reference and control systems, respectively, exhibit lower TPO expression and similar heterogeneity (Γ_anti-TPO_ and Γ_anti-IgG_ ∼ 280 and 330 µm^2^, respectively) to the system challenged with a rich desialylated platelet media. (*F*) Scatter plot of the individual secretion area from each individual cell within the population of the different systems after 180 min. The box indicates the 25th and 75th percentiles of the samples, respectively. TPO secretion from cells exposed to 99% and 8–9% desialylated platelets, respectively, was found to be κ_anti-TPO__+__desialylated_ _PL_ 17 µm^2^/h and κ_anti-TPO__+__sialylated_ _PL_ 13 µm^2^/h (*SI Appendix*). (*G*–*J*) Cell count histograms of the secretion area for each HepG2 cell population (including the control system functionalized with an antibody against IgG). The SD for each case is represented with a bar in each of the plots, which directly relates to the heterogeneity of the system.

We studied platelet-mediated regulation and secretion of TPO from exemplar human cells [HepG2 cell-line human hepatocyte carcinoma (*Methods*)] over a 500 × 500-µm^2^ region of interest. TPO secretion was monitored using a PCRS functionalized with anti-TPO and recorded as a function of exposure to human platelets with differing levels of desialylation (14) (*Methods*). Three exemplar systems were employed to fully evaluate platelet-mediated secretion of TPO by HepG2 cells (*Methods* and *SI Appendix*): (*i*) TPO secretion from HepG2 cells incubated with artificially aged platelets (anti-TPO + desialylated PL) (∼99% desialylation; *SI Appendix*). (*ii*) TPO secretion from HepG2 cells incubated with human platelets with naturally occurring levels of sialyation (anti-TPO + sialylated PL) (∼8–9% desialylation, *SI Appendix*). (*iii*) Inherent TPO secretion from HepG2 cells (anti-TPO). An additional control measurement involving a surface functionalized with anti-IgG was also performed to characterize the degree of nonspecific binding (anti-IgG). While HepG2 cells inherently secrete TPO (Fig. 3 *F* and *I*), the expression of TPO is consistently higher for HegG2 cells challenged with desialylated platelets (Fig. 3 *B*, *F*, and *G*). Not only do we observe up-regulation of TPO upon exposure to desialylated platelets, but we also see that TPO secretion is highly consistent between individual HepG2 cells (total of 30 cells). In contrast, TPO secretion from HepG2 cells challenged with naturally occurring levels of desialylated platelets (Fig. 3 *F* and *H*) displayed wide heterogeneity. We believe this reflects the heterogeneity of platelet age. Specifically, we find only 8–9% of platelets in the extracted sample are desialylated (*SI Appendix*) and are thus able to up-regulate TPO expression in the HepG2 cells. These measurements agree with the recent theory of TPO regulation which states that TPO levels are directly dependent on the concentration of circulating desialylated platelets. This validation highlights how our system can provide a simple, yet powerful approach for studying complex physiological systems in vitro at the single-cell resolution.

We have presented a real-time and label-free single-cell signaling analysis method based on photonic crystal resonant surfaces. The high spatial resolution of the method, coupled with the large field of view (whose dimensions are ultimately limited by the optical objective integrated in the system) and molecular specificity of the assay, enables high-throughput measurements of the dynamics of protein secretion from single cells within a cell population. These capabilities were used to examine and quantify the dynamics of cell-signaling heterogeneity within cell populations in vitro and in a timely manner appropriate for clinical applications. In particular, we were able to quantify TPO release at single-cell resolution and were able to show that TPO levels are directly dependent on the concentration of circulating desialylated platelets. Given its ease of use, simple computing implementation, and compatibility with standard inverted microscopes and other imaging techniques such as phase-contrast microscopy, we anticipate that the PCRS method will transform single-cell protein expression analysis from an experts-only technique into a broadly accessible single-cell signaling imaging methodology.

## Methods

### Photonic Crystal Resonant Imagining Surface.

The imaging surface consists of a grating etched into a 150-nm-thick silicon nitride (Si_3_N_4_) film on glass and is fabricated using electron beam lithography and reactive ion etching, which allows for multiple reuses after cleaning. For more detail, refer to our previous work (7, 15). First, the Si_3_N_4_ is cleaned in a piranha solution (1:3 hydrogen peroxide: sulfuric acid), rinsed in acetone and isopropanol, and dried with nitrogen. Next, the e-beam resist (ARP-09; AllResist GmbH) is spin-coated for 60 s at 3,000 rpm (Electronic Micro Systems Ltd.; EMS-4000), and soft baked on a hot plate at 180 °C for 10 min. For charge dissipation during e-beam exposure, a thin film of aluminum (∼20 nm) is deposited on top of the resist using a thermal evaporator (HEX; Mantis). The e-beam system (Voyager; Raith GmbH) exposes the resist with a base dose of 130 μC/cm^2^, after which it is developed for 2 min in Xylene, then rinsed with isopropanol and dried in N_2_. A sensor area of 2 × 2 mm is exposed per device, consisting of a grating with period of 555 nm and a fill factor of 80% to obtain a resonance wave centered around 840-nm wavelength. To transfer the grating into the Si_3_N_4_, it is etched with a CHF_3_:O_2_ gas mixture at 29:1 sccm, an rf power of 40 W, and a chamber pressure of 1.9 e^−1^ mbar, over 7-min etch time. Finally, to strip the remaining resist, the sample is sonicated in 1165 solvent (MicroChem) for 3 min, followed by a rinse in acetone and isopropanol, following a drying step with N_2_.

### Hyperspectral Imaging Measuring Setup.

A resonance image is formed by taking a sequence of bright-field images, each at a different illumination wavelength which is achieved by illuminating through a tunable filter (hyperspectral imaging). The resulting hyperspectral cube contains the spectrum from every pixel in the field of view. By analyzing the intensity values of each pixel, the resonance wavelength for each pixel can be determined. We fit the measured data from each pixel to a Fano resonance curve to accurately obtain the resonance wavelength with pm precision. Plotting the resonance wavelength of each pixel in the array then gives the resonance image. We repeat this process cyclically over time, being limited to a rate of 2.5 min per image by instrumental limitations (we note this can be improved to sub-30-s acquisition rates under appropriate conditions). The camera employed here is a CoolSnap Myo (Photometrics), and the objective lens is an Olympus NeoDplan 10× (N.A. = 0.25) which is the component that ultimately limits the field of view. The camera pixel size is 4.54 μm, and after magnification, each image pixel images a size of 0.925 μm. To provide some flexibility in matching the illumination wavelength to the resonance wavelength of the fabricated grating, we use a broadband laser (SM30; Leukos), combined with a custom-built grating-based monochromator to select a single illumination wavelength with a spectral width of 0.6 nm (0.25-nm wavelength step). To further improve the resolution of the images we interpolate the values between pixels down to low pm values. Images were captured using LabView, and image analysis and curve fitting were performed using MATLAB. The pixel fraction histogram to set the 1-nm threshold is obtained by dividing the total number of resonant pixels at a specific wavelength against the total number of pixels in the region of interest. We also incorporated a fluidic well for cell culturing made from acrylic, which is attached to the sensor chip to allow exposure to various analytes, while encapsulating the whole device setup inside an incubator set at 37 °C.

### Specific Surface Functionalization Toward TPO Proteins.

Si_3_N_4_ sensor chips are carefully cleaned before use, consisting of a 10-min cleaning step in a UV−ozone cleaner, followed by placing in solutions of Hellmanex III (2%) and ultrapure Milli-Q water (twice) and sonication in each bath for 10 min. The sensors are then dried with N_2_ gas and returned to the UV−ozone cleaner for 30 min, following an exposure to 15% nitric acid solution to reveal the oxide groups on the Si_3_N_4_ surface. Finally, the clean and hydroxyl-activated sensors are immediately transferred in a 3% vol/vol aminopropyltriethoxy silane solution in ethanol and left overnight. Finally, the chips are dried with N_2_ gas and a curing step is performed at 110 °C for 1 h, then loaded in the acrylic mount of the measuring setup (temperature controlled at 37 °C) and injection of Milli-Q water (*SI Appendix*). Oxidized 2% (20 mg/mL) dextran solution [Dextran T40 (40 kDa), 30 mM NaIO_4_; Sigma-Aldrich] is sequentially injected and left for 90 min (16), rinsed in Milli-Q water, further oxidized in 30 mM NaIO_4_ for 90 min, and rinsed again in Milli-Q water (*SI Appendix*). The surface is then rinsed in PBS (pH 7.4) and *Escherichia-coli*-derived animal-free recombinant human TPO (PeproTech) antibodies are thereafter injected at 50 μg/mL in PBS (pH 7.4) and left for 60 min. To prevent nonspecific binding from other biomolecules expressed from either BHK or HepG2 cell lines, casein-blocking agent is incorporated in the assay. We found an optimal concentration of 0.35× and 0.5× (in PBS pH 7.4) for BHK and HepG2 cell lines, respectively (*SI Appendix*), which were incubated for 45 min to complete the sensor functionalization. Once reaching this point, the sensor is washed with cell culture media (DMEM supplemented with 10% FBS, 1% Pen-Strep, and 25 mM Hepes; Gibco) before introducing the live cells.

### Cell Adsorption Kinetics Modeling.

Real-time expression of signaling molecules from single cells and their binding to the capture molecules of the functionalized sensor surface was modeled as a Langmuir adsorption isotherm by *assuming both processes as the same, global adsorption process. The equation to fit the secretion area of each cell per time unit is

$$\text{Secretion area}={\text{N}}_{0}\frac{\text{κ*t}}{1+\text{κ*t}},$$ [1]

with ${\text{N}}_{0}$ the offset of the area covered by the binding of signaling molecules in square micrometers, κ the Langmuir adsorption rate (square micrometers per hour), and t the time in hours. A least-squares regression analysis was used to find the best fit for our dataset, which yields a coefficient of determination R^2^ of 0.94 ± 0.04 for the 30 BHK-TPO cell population under study.

### BHK-TPO and HepG2 Cell Lines Culture.

BHK-TPO and HepG2 cells were cultured at 37 °C in a 5% CO_2_ incubator in DMEM supplemented with 10% FBS, 1% Pen-Strep, and 25 mM Hepes. Both BHK-TPO and HepG2 subconfluent cultures were kept at 2–4 × 10,000 cells per square centimeter. Cell monolayers were passaged following a rinse with 1× PBS (twice) and prewarmed (37 °C) TrypLE Express (Gibco) solution to cover the bottom of the flask, and incubated for 5 and 10 min, respectively. Once cells detached TrypLE Express was neutralized by adding 2× volume of complete growth medium.

### Isolation of Human Platelets from Whole Blood and Platelet Count.

Blood samples were obtained from healthy volunteers after informed consent and was approved by the Department of Biology Ethics Committee, University of York. Venous blood was collected from healthy volunteers and platelets were prepared following the protocol described in ref. 17. In short, using a 19-G butterfly needle, venous blood is taken into vacutainers and anticoagulated 5:1 with acid-citrate-dextrose (65 mM trisodium citrate, 70 mM citric acid, 100 mM dextrose, pH 4.4). Platelet-rich plasma is obtained by centrifugation at 100 × *g* for 20 min at room temperature. Platelets are then resuspended in wash buffer (150 mM NaCl, 20 mM Hepes, pH 6.5; both from Sigma-Aldrich) and centrifuged at 220 × *g* for 10 min at room temperature in the presence of 1 U/mL apyrase (Sigma-Aldrich) and 1 mM prostaglandin E1 (PGE1; Sigma-Aldrich). Finally, the platelet pellet is resuspended in Walsh’s buffer (137 mM NaCl, 20 mM PIPES, 5.6 mM dextrose, 1 g/L BSA, 1 mM MgCl_2_, 2.7 mM KCl, 3.3 mM NaH_2_PO_4_, pH 7.4; all from Sigma-Aldrich). Before any experimental procedure, platelets are left at room temperature for 30 min; after 30 min, most of the PGE1 has become inactive. Flow cytometry was employed to count the number of platelets per milliliter (*SI Appendix*).

### Platelet Desialylation.

Human platelets were treated with α2–3,6,8 neuraminidase from *Clostridium* *perfringens* (New England Biolabs) to remove terminal sialic acid (18). Isolated platelets in Walsh’s buffer (∼1.5 × 10^9^ platelets per milliliter) were incubated with 2.5 mU of α2–3,6,8 neuraminidase for 15 min at 37 °C. Desialylation was confirmed by lectin binding [biotinylated version of sambucus nigra (Vector Laboratories Ltd.)] via flow cytometry (*SI Appendix*).

## Supplementary Material

Supplementary File
