# Supplementary material for: Quantifying single-cell secretion in real time using resonant hyperspectral imaging
Source: Proc Natl Acad Sci U S A. 2018 Dec 10;115(52):13204–9. doi: 10.1073/pnas.1814977115 (PMC6310807; doi:10.1073/pnas.1814977115)
Supplement: Supplementary File [file pnas.1814977115.sapp.pdf]

SUPPORTING INFORMATION

Quantifying single cell secretion in real time using resonant hyperspectral imaging

José Juan-Colás[1,2*], Ian S. Hitchcock[3], Mark Coles[4], Steven Johnson[2] and Thomas F. Krauss[1]

[1] Department of Physics, University of York, Heslington, York YO10 5DD, UK
[2] Department of Electronic Engineering, University of York, Heslington, York YO10 5DD, UK
[3] Department of Biology, University of York, Heslington, York YO10 5DD, UK
[4] Kennedy Institute of Rheumatology, University of Oxford, Headington, Oxford OX3 7FY, UK
*Correspondence: (J.J-C) jose.juancolas@york.ac.uk

www.pnas.org/cgi/doi/10.1073/pnas.1814977115

Supplementary Figures

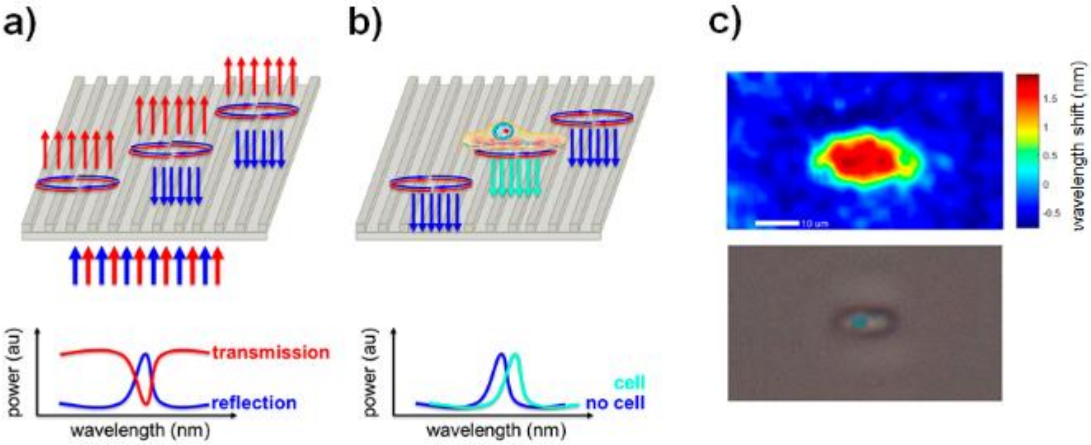

Supplementary Figure S1. Photonic crystal resonant imaging technique. a At resonance conditions, the resonant mode confined in the PCRS interferes destructively or constructively with the incoming light beam, creating either a dip (red trace) or a peak (blue trace) in the transmission and reflection sprectra, respectively. **b** Upon changes in the local refractive index, as those originated from cell attachment to the sensor surface, a resonance wavelength shift is induced (light blue trace), whose value is directly linked to the amount of biological matter. **c** Comparison between a photonic crystal resonant imaging hyperspectral image (top) and a phase-contrast microscopy (bottom) of a single Baby Hamster Kidney (BHK) cell attached to a PCRS. Higher resonance wavelength shifts are related to higher refractive index values, orginated from the presenc of both cell and expressed biomolecules. Secreted molecules are transparent and cannot be appreciated via phase contrast microscopy.

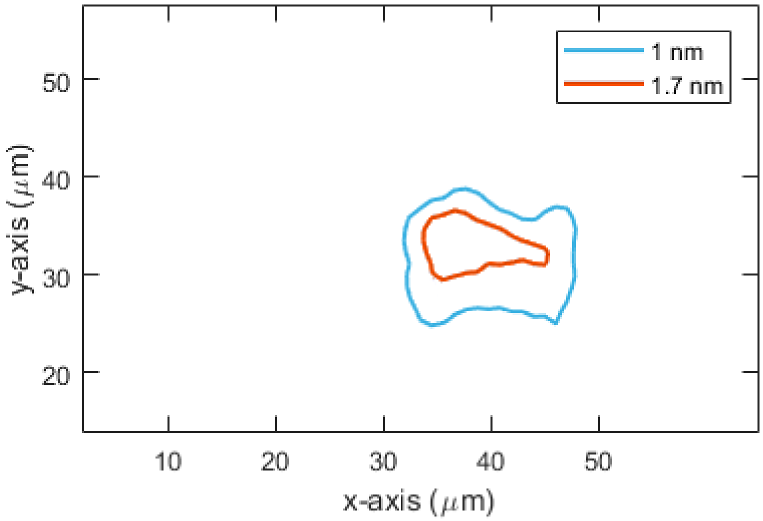

Supplementary Figure S2. Example of contour levels showing relative shift in resonance for protein secretion and cell attachment. Detailed analysis of the experimental data revealed that cell attachment leads to a wavelength shift over the area of each cell that is ≈ 1.7 ± 0.1 nm above the reference resonance wavelength The difference in the area delimited between the 1.7 and 1 nm contour levels (here exemplified for the cell shown in Figure 1g-h), respectively, delimits the secretion area of our measurements.

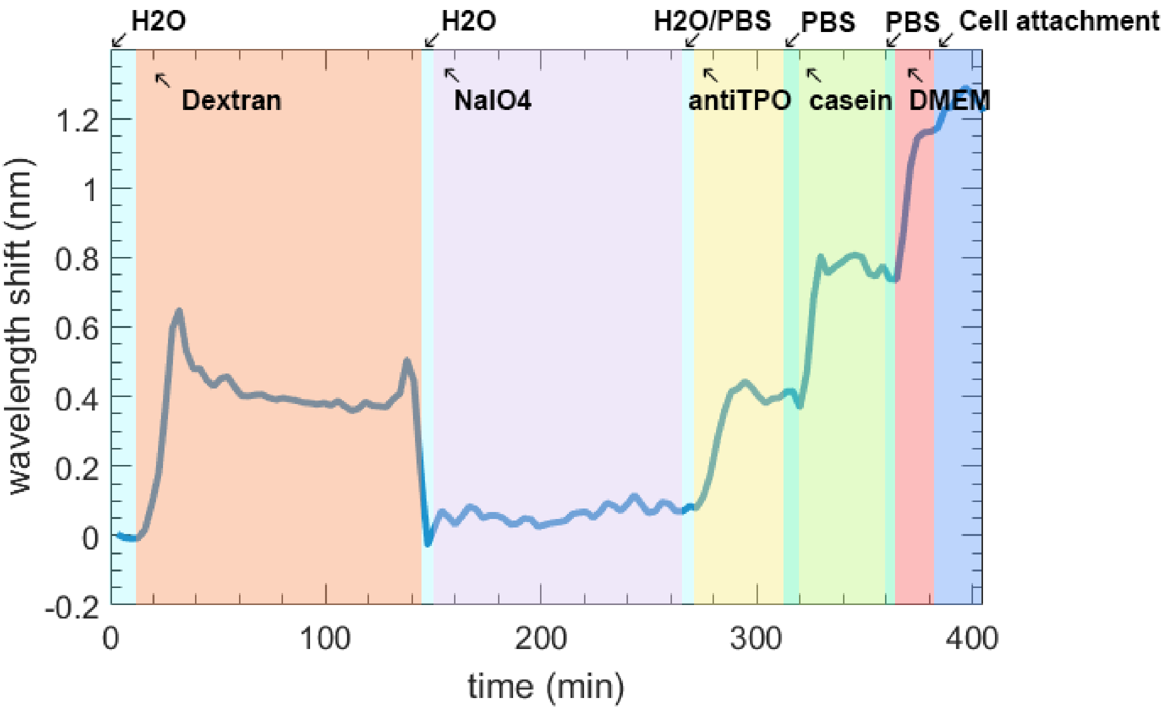

Supplementary Figure S3. Real-time TPO functionalisation of a PCRS. The PCRS APTES modified surface is firstly washed in Milli-Q water and then oxidized 2% (20 mg/mL) dextran solution (Dextran T40 (40 kDa), 30 mM $NaIO_4$, Sigma Aldrich) is sequentially injected and left for 90 min. It is then rinsed in Milli-Q water, further oxidized in 30 mM $NaIO_4$ for 90 min and rinsed again in Milli-Q water. The surface is then rinsed in phosphate-buffered saline (PBS, pH 7.4) and E.coli derived animal-free recombinant human TPO (PeproTech) antibodies are thereafter injected at 50 µg/mL in PBS (pH 7.4) and left for 40 min. Casein-blocking agent at a concentration of 0.35x (in PBS, pH 7.4) is incorporated in the assay, which is incubated for 40 min to complete the sensor functionalisation. Once reached this point, the sensor is washed with cell culture media (DMEM supplemented with 10% FBS, 1% Pen-Strep and 25 mM Hepes, Gibco) prior to introducing the live cells.

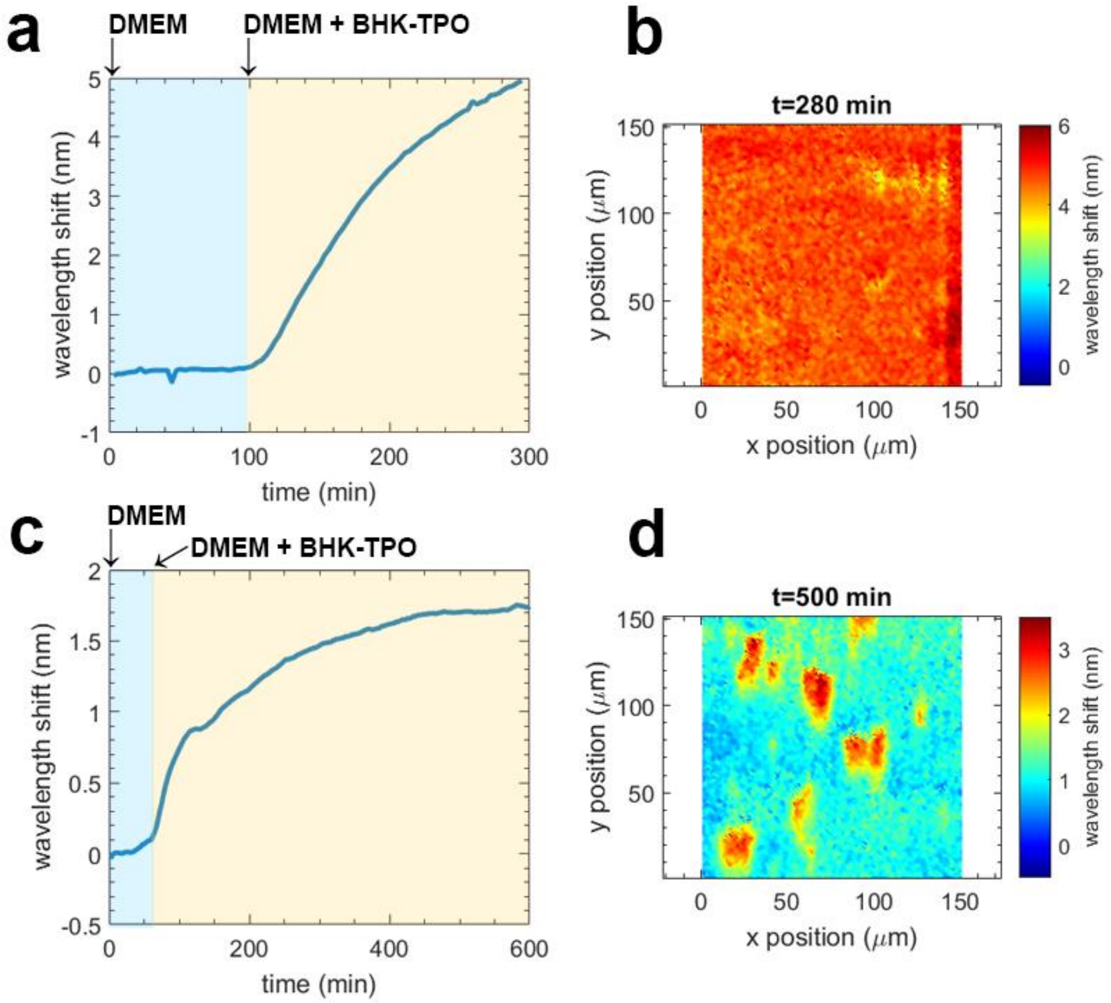

Supplementary Figure S4. BHK-TPO Blocking agent demonstration. a Average resonance wavelength shift over time upon addition of BHK-TPO cells on a PCRS without the addition casein-blocking agent (in a 22500 μm^2 region of interest). **b** No cells can be observed after 280 min as the refractive index shift they produce is masked by the non-specific binding. **c** Average resonance wavelength shift over time upon addition of BHK-TPO cells on a PCRS with 0.35x casein-blocking agent (in a 22500 μm^2 region of interest). **d** Cells can be clearly identified after 500 min as the non-specific binding in minimised due to the presence of the casein-blocking agent.

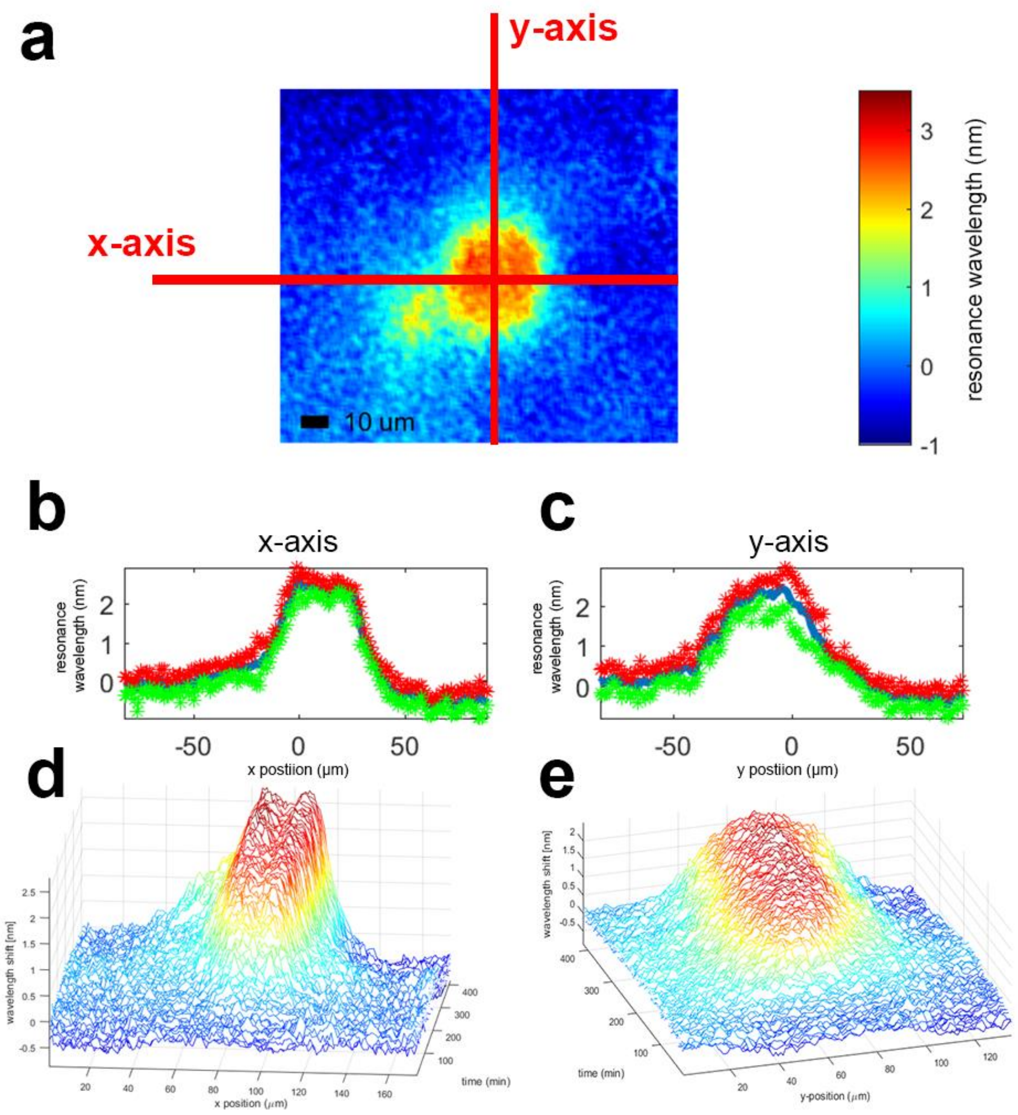

Supplementary Figure S5. Real-time mapping of the secretion gradient of a BHK-TPO cell. a Exemplar hyperspectral image of a BHK-TPO cell on a PCRS at t=100 min. The resonance wavelength is measured along two planes which are aligned with the x and y axis, respectively. The region in red delimits the size of the plane, which has a width of 3 pixels (~2.5 µm). **b-c** Distribution of the resonance wavelength shift at t=100 min over the x and y planes, respectively. The section of the curve which exhibits a resonance wavelength sift above ~ 0.5 nm indicates the presence of the cell. The blue trace represents the average value in the delimited area, while the red and the green dots indicate the maximum and the minimum values inside this area, respectively. **d-e** Evolution of the change in resonance wavelength over time due to the presence of the cell and the TPO secreted around the cell.

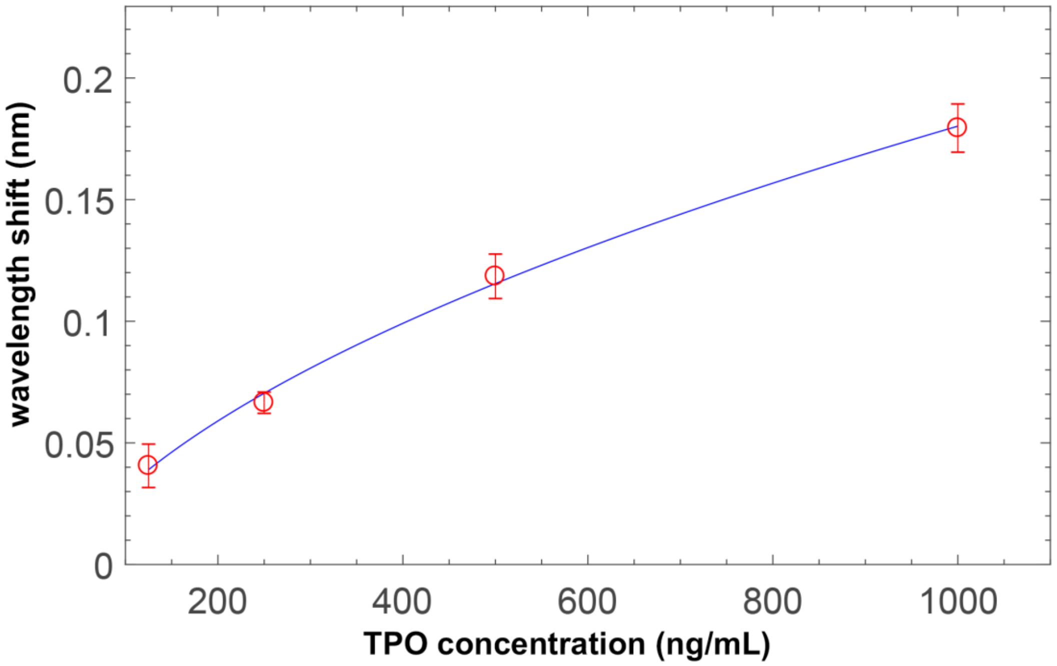

Supplementary Figure S6. Recombinant TPO protein detection. The PCRS surface was functionalised with anti-TPO (see Methods) to detect different concentrations of suspended recombinant Human TPO (Peprotech) in DMEM (supplemented with 10% FBS, 1% Pen-Strep and 25 mM Hepes, Gibco). The protein was incubated on the sensor surface for 20 min at 125, 250, 500 and 1000 ng/mL concentrations, followed by a vigorous PBS rinse (twice, pH 7.4) between steps. The red circles represent the average value during the last 5 minutes of each concentration level, while bars represent the standard deviation of the resonance wavelength over these 5 minutes. We note that, recombinant TPO has a significantly lower molecular weight (18.6 KDa) in comparison to native TPO expressed by both BHK-TPO and HepG2 cells (85 kDa), and therefore binding of recombinant TPO to the functionalized PCRS induces lower refractive index changes than binding of native TPO protein.

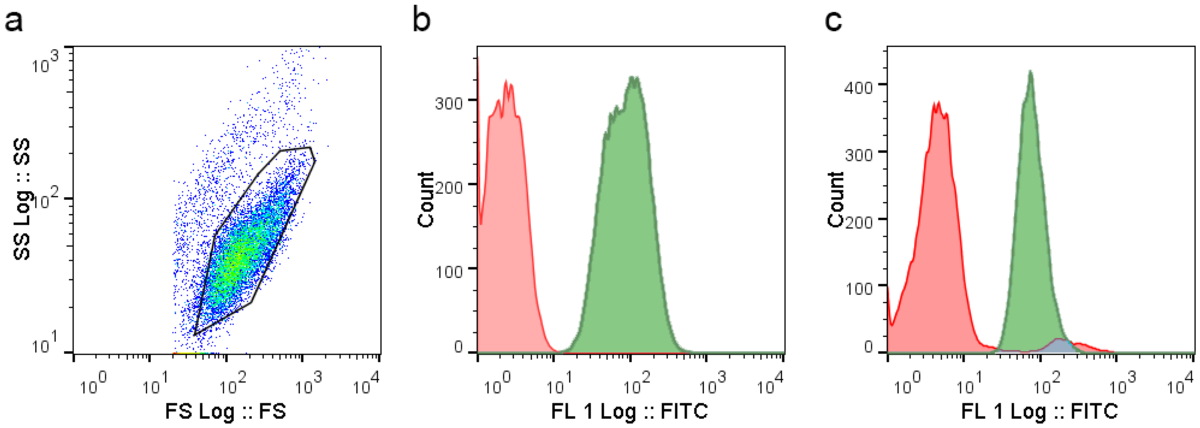

Supplementary Figure S7. Flow cytometric determination of desialylation levels in isolated human platelets. a Isolated platelets from plasma (see Methods) are labelled with FITC anti-human CD41a (BD Bioscience), found at 2.3 x 10^6 counts/mL (measured using CytoFLEX S, Beckman Coulter). **b** Platelet exposed to streptavidin modified Cy5 label (BD Bioscience) and CD41a-labeled platelets are both normally distributed between 1 to 10 FITC (red), and 10 to 1,000 FITC (green), respectively. **c** Both desialylated and sialylated platelets are treated with biotinylated SMA (control lectin, see Methods) to quantify desialylation levels. They are then Cy5-labeled through conjugation with streptavidin modified Cy5 label. Cy5-labeled desialylated platelets are normally distributed around 10^2 FITC (green). In contrast, non-treated Cy5-labeled platelets are primordially normally distributed between 1 and 10 FITC (red). The desialylation level is measured by integrating the counts from 20 to 10^4 FTIC and normalised over the total count number. A ~99% desialylation level is found for the α2-3,6,8 neuraminidase treated (desialylated) sample (green), while just 8-9% is found on the non-treated one (red).

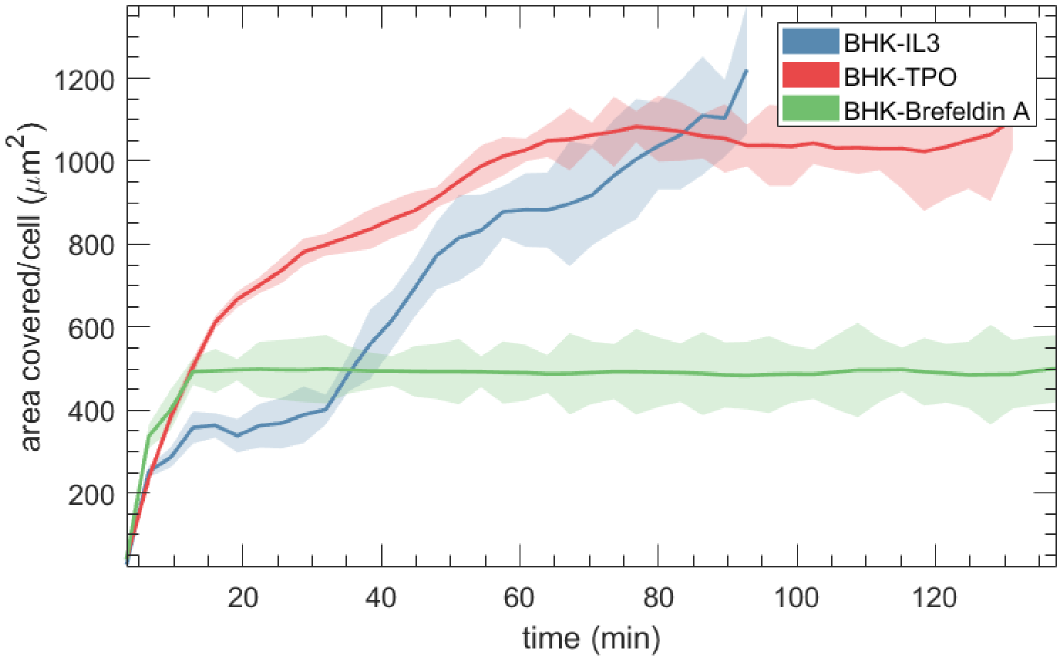

Supplementary Figure S8. Secretion dynamics comparison between three 30 cell populations of BHK-IL3, BHK-TPO and BHK-TPO treated with Brefeldin A. To monitor the secretion from transfected BHK cells to over express Interleukin 3 (IL3), the surface of the PCRS was functionalised similarly as done for TPO but swapping the anti-TPO with anti-IL3 (see Methods). To render the PCRS reactive to IL3, anti-Murine IL3 (Peprotech) was incubated of the PCRS device at 50 µg/mL in PBS (pH 7.4) and for 60 min. The dynamics of the different protein secretion from the three 30 cell populations was characterised over a region of interest of 250000 μm^2 in real-time, showing how our method is generic to diverse cell populations and systems.

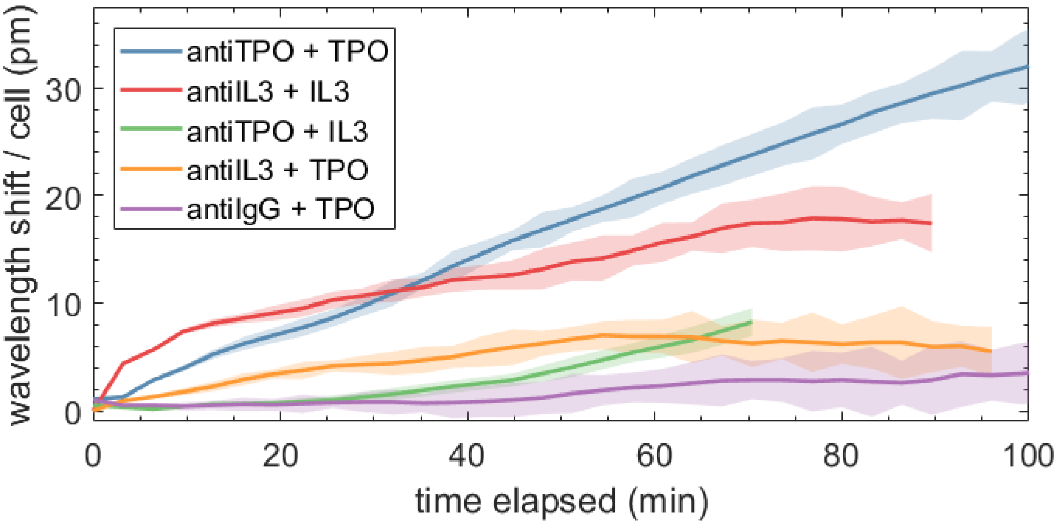

Supplementary Figure S9. Characterisation of non-specific binding. The specifity of our method was validated by running a series of control experiments where different combinations of antibody-secreted proteins were employed. Five different experiments were carried out: two for specific antibody-secretion protein pairs, and three for non-specific pairs. In all five experiments, the surface was functionalised using the antibody indicated in the legend of the graph and following the protocol described in the methods section. Similarly, cells were incubated on the functionalised PCRS surface in cell culture media as described in the methods section. A region of interest of ~200000 μm^2 was used, and the overall wavelength shift in this region of interest was normalised by the number of cells present to calibrate the result. We found significant difference in both the binding kinetics and overall wavelength shift between the specific (anti-TPO + TPO, antiIL3 + IL3) and the non-specific (anti-TPO + IL3, anti-IL3 + TPO and anti-IgG + TPO), which demonstrate the specificity of our assay.

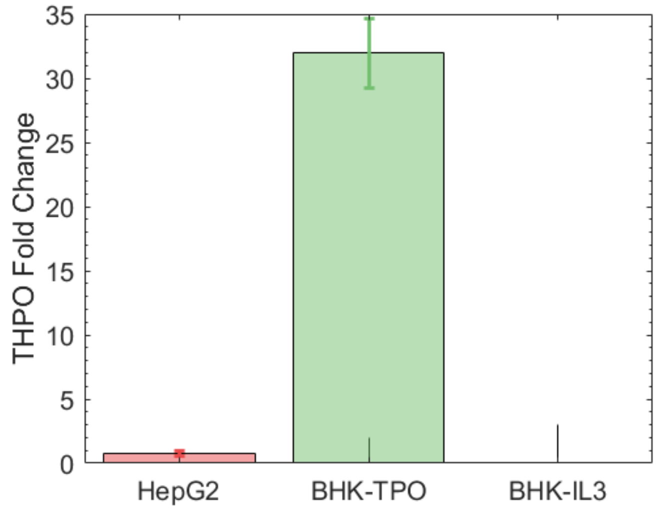

Supplementary Figure S10. qPCR analysis of THPO gene expression relative to HepG2 cells. Bars show mean values ± SEM, P****<0.0001. Unpaired T Test was used. N=3. Total RNA was extracted from BHK-TPO, BHK-IL3 or HepG2 cells using an RNeasy Mini Kit (Qiagen) following the manufacturer's protocol. RNA was reverse transcribed using a High-Capacity cDNA Reverse Transcription Kit (Applied Biosytems). Gene expression was analysed using Taqman Assays (Applied Biosytems): primers and probes used were THPO (Hs01061346_m1) and 18S rRNA (4310893E), whilst the mastermix was Taqman Fast Advanced MasterMix. 18S rRNA was used to normalise the amount of cDNA in each reaction. Amplification of cDNA was performed using the Applied Biosytems 7500 Fast Real-Time PCR System. Relative THPO expression was calculated as fold change= $2^{-\Delta\Delta C_T}$ (1).

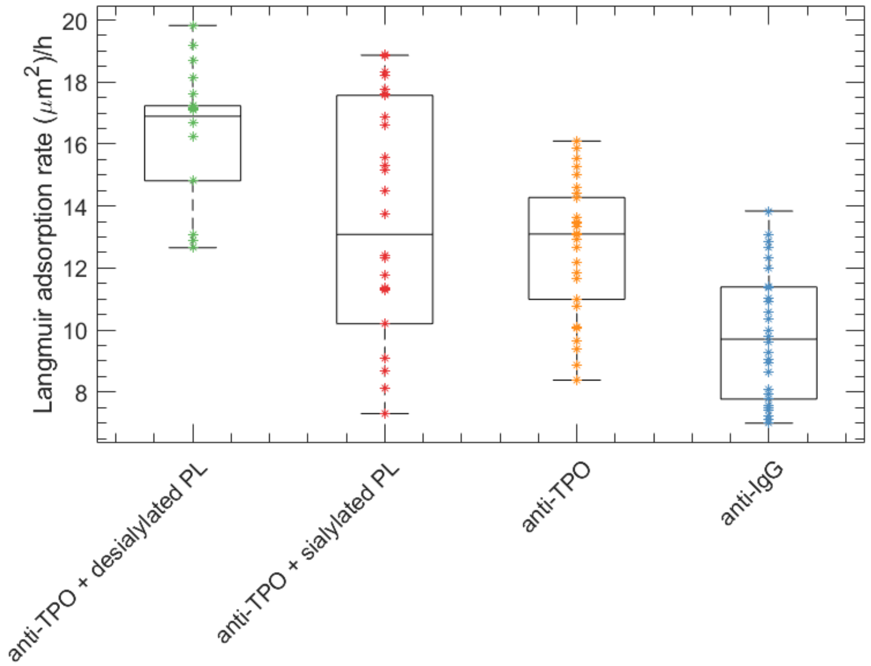

Supplementary Figure S11. TPO secretion rate from HepG2 cells. We modelled the Langmuir rate constant associated with each HepG2 cell system to deconvolute the influence of the inherent cell attachment area from the measurement. We obtained rates of ~ 17, 13, 13.5 and 9 $\mu m^2/h$ for the for HegG2 cells challenged with desialylated platelets, sialylated platelets, non-challenged and non-challenged with control antibody on surface, respectively. Coefficients of determination R^2 of 0.88 ± 0.08, 0.5 ± 0.11, 0.7 ± 0.07 and 0.65 ± 0.09 were respectively obtained.

Supplementary References

1. Livak KJ, Schmittgen TD (2001) Analysis of relative gene expression data using real-time quantitative PCR and the 2-ΔΔCT method. *Methods* 25(4):402–408.
